# The Usefulness of [^18^F]F-Fluorodeoxyglucose and [^18^F]F-Sodium Fluoride Positron Emission Tomography Imaging in the Assessment of Early-Stage Aortic Valve Degeneration after Transcatheter Aortic Valve Implantation (TAVI)—Protocol Description and Preliminary Results

**DOI:** 10.3390/jcm10030431

**Published:** 2021-01-22

**Authors:** Danuta Sorysz, Rafał Januszek, Anna Sowa-Staszczak, Anna Grochowska, Marta Opalińska, Maciej Bagieński, Barbara Zawiślak, Artur Dziewierz, Tomasz Tokarek, Agata Krawczyk-Ożóg, Stanisław Bartuś, Dariusz Dudek

**Affiliations:** 1 Department of Cardiology and Cardiovascular Interventions, University Hospital, 30-688 Kraków, Poland; maciejbagienski@gmail.com (M.B.); zawislak.barbara@gmail.com (B.Z.); adziewierz@gmail.com (A.D.); tomek.tokarek@gmail.com (T.T.); krawczyk.ozog@gmail.com (A.K.-O.); stanislaw.bartus@uj.edu.pl (S.B.); mcdudek@cyfronet.pl (D.D.); 2 Department of Clinical Rehabilitation, University of Physical Education, 31-571 Kraków, Poland; 3 Nuclear Medicine Unit, Department of Endocrinology, University Hospital, 31-501 Kraków, Poland; sowiana@gmail.com (A.S.-S.); mkal@vp.pl (M.O.); 4 Department of Endocrinology, University Hospital, Jagiellonian University Medical College, 31-501 Kraków, Poland; 5 Department of Radiology, University Hospital, Jagiellonian University Medical College, 31-501 Kraków, Poland; agrochowska@su.krakow.pl; 6 Department of Cardiology, Jagiellonian University Medical College, 31-008 Kraków, Poland

**Keywords:** degeneration, PET/CT imaging, TAVI durability

## Abstract

Transcatheter aortic valve implantation (TAVI) is now a well-established treatment for severe aortic stenosis. As the number of procedures and indications increase, the age of patients decreases. However, their durability and factors accelerating the process of degeneration are not well-known. The aim of the study was to verify the possibility of using [^18^F]F-sodium fluoride ([^18^F]F-NaF) and [^18^F]F-fluorodeoxyglucose ([^18^F]F-FDG) positron emission tomography/computed tomography (PET/CT) in assessing the intensity of TAVI valve degenerative processes. In 73 TAVI patients, transthoracic echocardiography (TTE) at initial (before TAVI), baseline (after TAVI), and during follow-up, as well as transesophageal echocardiography (TEE) and PET/CT, were performed using [^18^F]F-NaF and [^18^F]F-FDG at the six-month follow-up (FU) visit as a part of a two-year FU period. The morphology of TAVI valve leaflets were assessed in TEE, transvalvular gradients and effective orifice area (EOA) in TTE. Calcium scores and PET tracer activity were counted. We assessed the relationship between [^18^F]F-NaF and [^18^F]F-FDG PET/CT uptake at the 6 = month FU with selected indices e.g.,: transvalvular gradient, valve type, EOA and insufficiency grade at following time points after the TAVI procedure. We present the preliminary PET/CT ([^18^F]F-NaF, [^18^F]F-FDG) results at the six-month follow-up period as are part of an ongoing study, which will last two years FU. We enrolled 73 TAVI patients with the mean age of 82.49 ± 7.11 years. A significant decrease in transvalvular gradient and increase of effective orifice area and left ventricle ejection fraction were observed. At six months, FU valve thrombosis was diagnosed in four patients, while 7.6% of patients refused planned controls due to the COVID-19 pandemic. We noticed significant correlations between valve types, EOA and transaortic valve gradients, as well as [^18^F]F-NaF and [^18^F]F-FDG uptake in PET/CT. PET/CT imaging with the use of [^18^F]F-FDG and [^18^F]F-NaF is intended to be feasible, and it practically allows the standardized uptake value (SUV) to differentiate the area containing the TAVI leaflets from the SUV directly adjacent to the ring calcifications and the calcified native leaflets. This could become the seed for future detection and evaluation capabilities regarding the progression of even early degenerative lesions to the TAVI valve, expressed as local leaflet inflammation and microcalcifications.

## 1. Introduction

Aortic stenosis (AS) is currently the most common type of valve disease. The increase in frequency of the degenerative form is associated with aging, and transcatheter aortic valve implantation (TAVI) has become the standard treatment in the population at increased risk of surgery. Due to the gradual expansion of indications and the performance of TAVI in moderate-risk patients, often those younger, the problem of their durability and the factors influencing them has become urgent, especially within the context of results concerning the latest multicenter trials in low-risk patients [1]. The problem of bioprosthetic valve degeneration after TAVI procedure is little-known, in comparison to standard surgical bioprosthetic aortic valve replacement [2]. It is also intriguing to learn about the starting point, mechanisms and risk factors for their formation, and potential ways of slowing down this process. Therefore, in the presented study, we aim to confirm the utility of positron emission tomography/computed tomography (PET/CT) in the evaluation TAVI valve degeneration processes, with insight into their inflammatory and calcification mechanisms.

## 2. Methods

**Figure 1:**
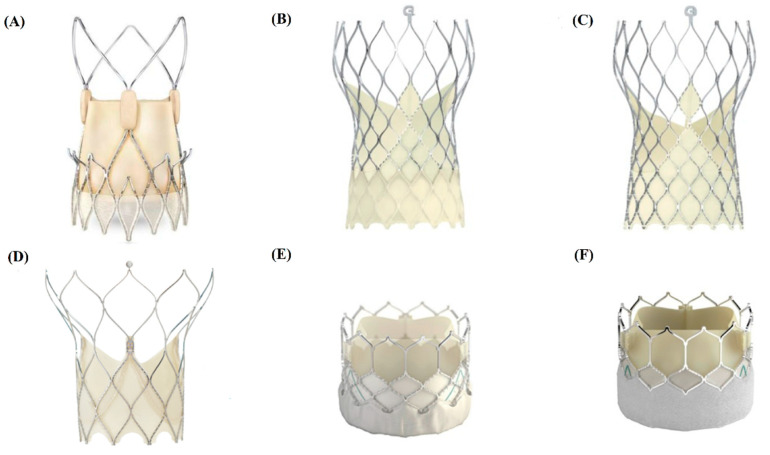
TAVI valves included in the current study. (**A**) Acurate Neo (Boston Scientific SciMed Inc, Maple Grove, MN, USA); (**B**) Evolut PRO (Medtronic, Minneapolis, MN, USA); (**C**) Evolut R (Medtronic, Minneapolis, MN, USA); (**D**) Portico^®^ valve (St. Jude Medical, St. Paul, MN, USA); (**E**) SAPIEN 3 (Edwards Lifesciences, Irvine, CA, USA); (**F**) SAPIEN 3 Ultra (Edwards Lifesciences, Irvine, CA, USA).

This research is designed as a prospective, observational, single-center study. There is no planned control group. To date, we have enrolled 73 consecutive patients with symptomatic, severe AS, qualified for the TAVI procedure with different types of bioprosthetic valves (Figure 1).

The protocol of our research includes initial visit (2 days before TAVI procedure) and baseline visit (up to one month after TAVI procedure), as well as at 6-, 1

2- and 24-month follow-up (FU) visits, accompanied by the transthoracic echocardiography (TTE) examination. The analysis presented in the current manuscript covers data up to the sixth month of the FU period. PET/CT and transesophageal echocardiography (TEE) were performed at the 6-month visit and this was recognized as the baseline visit for the two examinations, while the final PET/CT and TEE will be performed at the 24-month visit, according to the prespecified protocol. Blood samples are to be collected before TAVI (initial visit), at 6 months and 24 months after TAVI. The assessment of blood level proinflammatory and calcification markers is planned after completing all FU visits and collecting all blood samples.

### 2.1. Echocardiography

**Figure 2:**
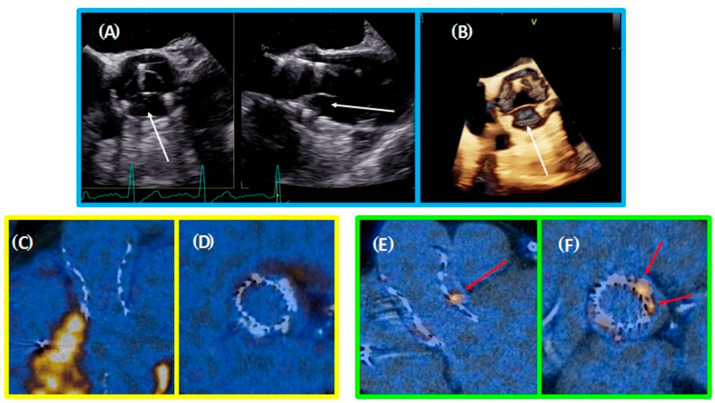
**Blue frame:** Transesophageal echocardiography (**A**) multi-D reconstruction: Acurate Neo valve with thrombotic leaflet (white arrows); (**B**) three dimensional reconstruction: Acurate Neo Valve with marked thrombotic leaflet (white arrow); **Yellow frame:** [^18^F]F-fluorodeoxyglucose positron emission tomography/computed tomography—no visible uptake at the level of the leaflets; (**C**) TAVI valve long axis, (**D**) TAVI valve short axis; **Green frame:** [^18^F]F-sodium fluoride positron emission tomography/computed tomography- red arrows: visible local uptakes out of the valve (aortic annulus calcifications); (**E**) TAVI valve long axis; (**F**) TAVI valve short axis.

All patients underwent TTE, including M-mode, two-dimensional, three-dimensional and Doppler imaging at the initial visit (before TAVI procedure) and during FU visits, including that at baseline (Figure 2A,B).

Close attention was paid to all acquisition settings in order to maximize image quality. For better visualization of TAVI valve leaflets two-dimensional and three-dimensional TEE was completed at the 6-month FU. All TTE and TEE examinations were performed using the Vivid E9 (GE Healthcare, Waukesha, WI, USA). The post-processing evaluation was carried out using a dedicated workstation (EchoPAC, GE Healthcare, Waukesha, WI, USA). The linear measurements were taken using virtual calipers. TTE examinations and measurements were performed according to the recommendations for assessment of aortic stenosis and TAVI valves [3,4]. TEE- visual assessment of AVC degree was done using a previously proposed semi-quantitative 5-grade scoring system for echocardiographic images [5]:

1. Normal leaflets without thickening or calcification;
2. Evidence of thickening but without calcification;
3. Calcification: small calcium spot not exceeding one-third of the leaflet area;
4. Moderate calcification: calcium not exceeding two-thirds of the leaflet area;
5. Heavily calcified: calcification covering more than two-thirds of the leaflet area.

### 2.2. Positron Emission Tomography/Computed Tomography (PET/CT)

Electrocardiography (ECG)-gated PET/CT scans of the aortic valve were performed with the use of [^18^F]F-fluorodeoxyglucose (FDG) and [^18^F]F-sodium fluoride (NaF) on GE DISCOVERY 690 VCT scanner (GE Healthcare, Milwaukee, WI, USA) (Figure 2). In the case of [^18^F]F-FDG, a target dose of 4 MBq per kg of body mass was injected intravenously, and patients rested in a quiet environment for 60 min. An attenuation-correction CT scan (nonenhanced, ECG-gated, low dose 120 kV with modulated rays (Smart mA) range 80 to 220 mAs) was performed, followed by PET imaging covering 1 bed position (15-cm long) centered over the valve in 3-dimensional mode for 16 min. The [^18^F]F-NaF was injected intravenously in the dose of 4 MBq per kg of body mass and acquisition was performed as described above. ECG-gated was carried out for the CT module in both study protocols.

The PET data were reconstructed with the use of the GE (matrix size 128 × 128, Vue Point FX reconstruction method: 24 subsets, 2 iterations with Cardiac 3D filter, 12-mm cut off for static image and Vue Point FX reconstruction method: 24 subsets, 5 iterations with Cardiac 3D filter, 4.3 mm-cut off for gated image) reconstruction algorithm. Corrections were applied for attenuation, dead time, scatter and random coincidences. All image analyses were performed on fused PET/CT data sets. Slice thickness of the PET short-axis images was 3.3mm.

**Figure 3:**
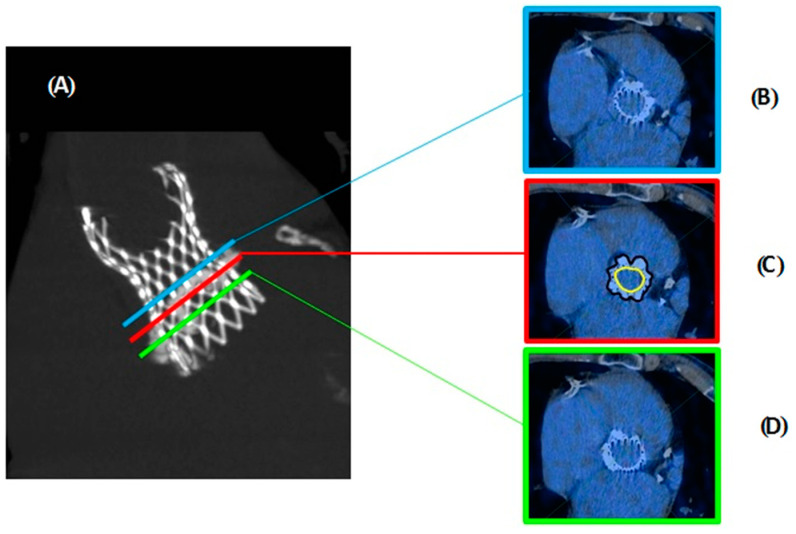
The methodology of leaflet ROI measurement in PET/CT examinations (**A**)—TAVI Evolut R Valve on CT—image used for estimation of leaflets localization and the choice of ROI levels. The top, middle, bottom cross-section of value used for measurements were marked with colors; Cross-sections of the valve on PET/CT examination (**B**)—on the top level (blue color); (**C**)—middle level (red color), yellow and black contour show method of inner and outer outline (ROI) for SUV measurements; (**D**)—bottom level (green color).

For both [^18^F]F-NaF and [^18^F]F-FDG PET/CT scans, the measurements were executed using the same method as described below (Figure 3). Due to use of ECG-gated PET/CT scans to increase the chance of more reliable results, the measurements were taken when the valve leaflets had the highest chance of being closed. Prior to performing any measurements, the heart/valve was rotated in 3-dimensional multiplanar reconstruction mode to visualize the valve from the coaxial short-axis view. This facilitated the more precise delineation regarding regions of interest (ROI): Leaflets of the valve and aortic annulus. In all cases, as a reference values mediastinal blood pool structures (MBPS) on descending aorta, maximal standardized uptake value (SUV) and SUV mean and mean density in Hounsfield units were measured. In regards to TAVI the first measurements were performed at the level of the closed leaflets (information provided by CT stent visualization and manufacturers of implanted TAVI valves). The regions of interest (according to stent shape, called middle ROI) inside and outside of the stent were drawn. Next, ROIs were drawn on two adjacent slices (one slice above and below, called later upper and bottom) to increase the chance of whole valve examination. The mean and maximum SUVs were calculated for each slice and then for the valve, mean value was calculated, subtraction SUV values of the regions of interest and MBPS SUV were analyzed according to suggestions in previously published papers [6] (Figure 3). MBPS SUVs were derived from circular regions of interest drawn in the central blood pool of the descending aorta at the same level as the artificial valve was visible. This enabled the mean and maximum tissue-to-background ratio (TBR) counting [6]. Those counts reduce the impact of individual SUV (max and mean variability) on the result. All study patients provided their written informed consent to participate the study, which was approved by the local Ethics Committee.

### 2.3. Statistical Analysis

Categorical variables are presented as numbers and percentages. Continuous variables are expressed as mean (standard deviation, (SD)) and median (interquartile range, (IQR)). Normality was assessed via the Shapiro-Wilk test. Equality of variance was evaluated using Levene’s test. The differences between the two groups were compared using the Student’s or Welch’s *t*-test, depending on the equality of variances for normally distributed variables. The Mann-Whitney U test was applied for non-normally distributed continuous variables. Categorical variables were compared with Pearson’s chi-squared or Fisher’s exact test if 20% of cells had an expected count of less than 5 (Monte Carlo simulation for Fisher’s test using tables of higher dimensions than 2 × 2). Spearman’s linear correlation test was used to assess mutual relationships between selected indices. Statistical analysis was performed using JMP, version 15.2.0 (SAS Institute Inc., Cary, NC, USA, 2020).

## 3. Results

### 3.1. General Characteristics

We present the results the 6-month follow-up after TAVI procedure including TTE, TEE, [^18^F]F-NaF-PET/CT and [^18^F]F-FDG-PET/CT. Initial clinical characteristics (before TAVI procedure) are presented in Table 1, while initial and FU echocardiographic indices are placed in the Table 2.

In the current study, we have had 6 types of aortic valves, which were used for implantation during the TAVI procedure (Figure 1). Among them, there were 55.9% of patients with the Evolut R valve (Medtronic, Minneapolis, MN, USA), 16.9% patients with the Acurate Neo valve (Boston Scientific SciMed Inc, Maple Grove, MN, USA), 11.9% patients with the SAPIEN 3 valve (Edwards Lifesciences, Irvine, CA, USA), 6.8% patients with the Evolut PRO valve (Medtronic, Minneapolis, MN, USA), 6.8% patients with the Portico^®^ valve (St. Jude Medical) and 1.7% patients with the SAPIEN 3 Ultra valve (Edwards Lifesciences, Irvine, CA, USA).We observed 9.6% 1-month and 13.7% 6-month mortality (10 pts) rates among patients who completed the FU. Asymptomatic valve thrombosis was diagnosed at the 6-month FU in 9.6% of patients, and for this reason, PET/CT examination was postponed until the thrombotic lesions resolved. Among the patients, 7 refused to take part in the 6-month FU on site visit because of the SARS-CoV-19 pandemic.

### 3.2. Echocardiography

We observed a significant decrease in maximal and mean transvalvular gradient and an increase in effective orifice area as well as ejection fraction after TAVI (Table 2). During the 6-month FU, we did not observe significant changes in valvular parameters (Table 2). The 6-month FU TEE showed thickening and slight reduction of leaflet motion in 6 patients, but the anticoagulation had resolved these abnormalities. Score evaluation was performed on the control TEE. Considering the calcification scoring system, all patients at 6 months of FU presented grade 1 on this scale. Thrombosis of TAVI valve was noticed in 1 patient with the Evolut R valve and two patients the with Edwards Sapien 3 valves at 6 months of FU.

### 3.3. [18. F]F-FDG and [^18^F]F-NaF PET/CT

#### 3.3.1. Differences in [^18^F]F-FDG and [^18^F]F-NaF Uptake between Inner and Outer Areas of Particular Levels of TAVI Valves

The term of inner area is defined as the area lying within the valve (including its leaflets but not the valve stent and aortic annulus), the outer area is defined as the area that includes its leaflets and aortic annulus. When considering the [^18^F]F-NaF marker, there were significant differences between the inner and outer areas for maximal SUV uptake on the top (*p* = 0.0001), middle (*p* = 0.001), bottom (*p* = 0.004) mapping ROI (region of interest), as well as for maximal SUV uptake corrected by MBPS for top (*p* = 0.0008), middle (*p* = 0.01) and bottom (*p* = 0.01) mapping levels (Table 3). Maximal [^18^F]F-FDG SUV uptakes were significantly different between inner and outer areas for top (*p* = 0.04), middle (*p* = 0.01) and bottom (*p* = 0.004) mapping levels. Maximal [^18^F]F-FDG SUV uptakes corrected by MBPS were no longer significantly different between the inner and outer assessment areas for top, middle and bottom mapping levels (Table 3).

There were no significant differences between the inner and outer assessment areas for mean [^18^F]F-NaF SUV and [^18^F]F-NaF SUV uptakes corrected by MBPS, independently of the mapping level (Table 3).

#### 3.3.2. Differences between Particular Levels for [^18^F]F-FDG and [^18^F]F-NaF Uptake Regarding the Averaged Inner and Outer Analyzed Areas of TAVI Valves

There were no significant differences when comparing the middle and top levels for [^18^F]F-FDG and [^18^F]F-NaF uptakes. There were also no significant differences when comparing the bottom and top segments of [^18^F]F-FDG and [^18^F]F-NaF uptakes, or bottom and middle segments. Moreover, there were no statistically significant differences in the comparison all of 3 assessed segments (bottom, middle and top) for [^18^F]F-FDG and [^18^F]F-NaF uptakes.

### 3.4. Correlations

#### 3.4.1. Effective Orifice Area

**Figure 4:**
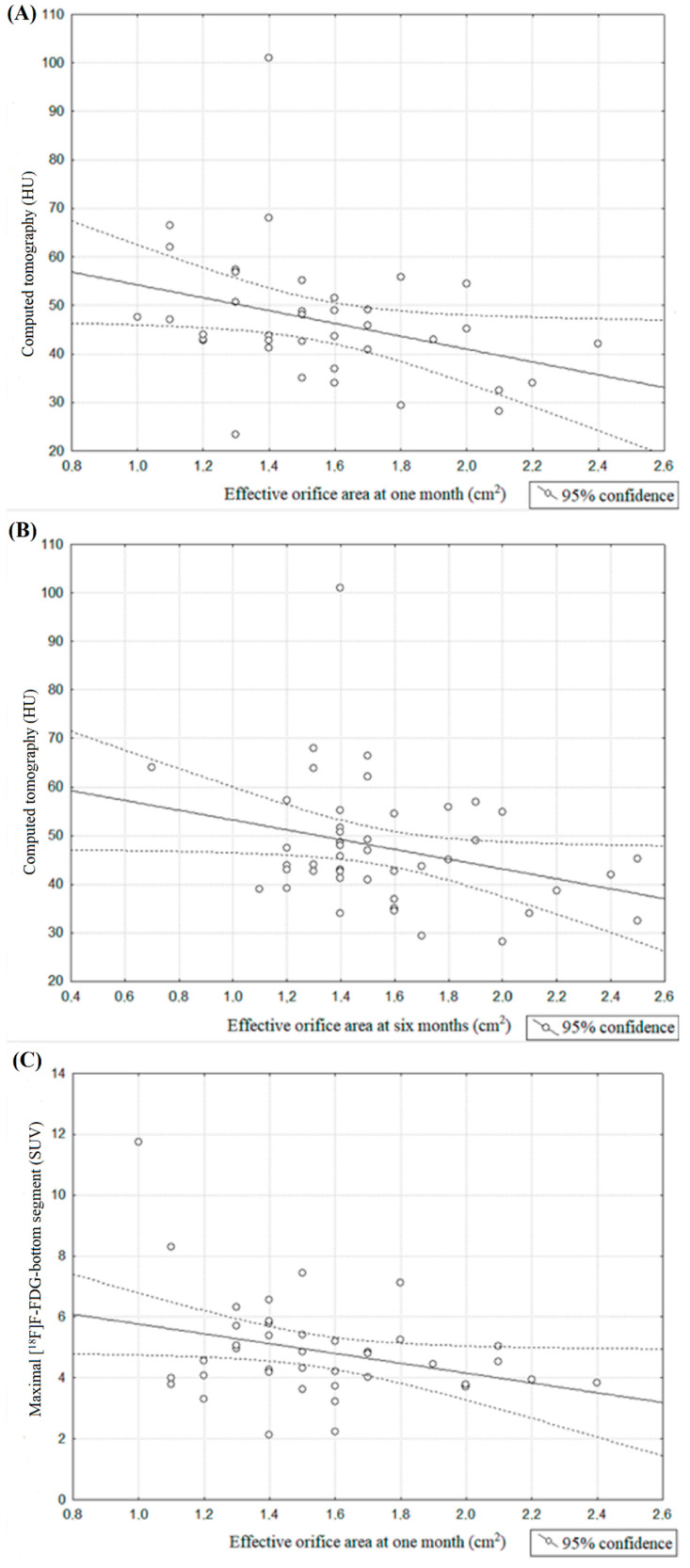
(**A**) Spearman’s correlation coefficient between effective orifice area at 1 month and [^18^F]F-fluorodeoxyglucose (HU) assessed at 6 month; (**B**) Spearman’s correlation coefficient between effective orifice area at 6 months and [^18^F]F-fluorodeoxyglucose (HU) assessed at 6 month; (**C**) Spearman’s correlation coefficient between effective orifice area at 1 month and maximal [^18^F]F-fluorodeoxyglucose assessed for bottom segment assessed at 6 months of FU period.

We noticed significant correlations between the effective orifice area (EOA) assessed by TTE at 1 month and Hounsfield units assessed by CT at 6 months of FU (*r* = −0.32, *p* = 0.04; Figure 4A) and with EOA measured at 6 months (*r* = −0.29, *p* = 0.04; Figure 4B). Also there was a borderline correlation between EOA measured at 1 month and maximal [^18^F]F-NaF of the bottom segment assessed by PET/CT at 6 months of FU (*r* = −0.31, *p* = 0.05; Figure 4C). There were no significant correlations for EOA measured at 1 or 6 months and other PET/CT indices.

#### 3.4.2. Aortic Valve Gradients

**Figure 5:**
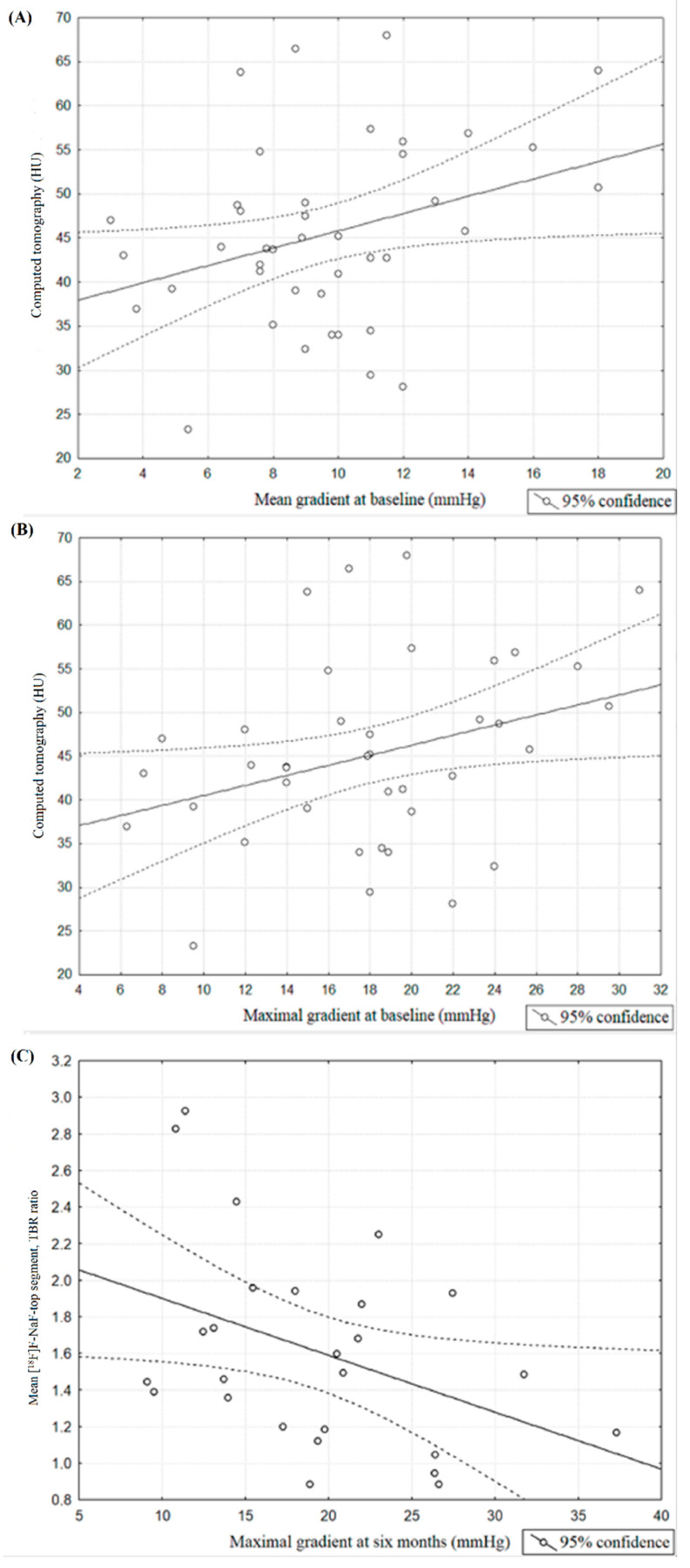
(**A**) Spearman’s correlation coefficient between mean aortic valve gradient measured at baseline and [^18^F]F-fluorodeoxyglucose (HU) measured at 6 months after TAVI procedure. (**B**) Spearman’s correlation coefficient between maximal aortic valve gradient measured at baseline and [^18^F]F-fluorodeoxyglucose (HU) measured at 6 months after TAVI procedure (**C**) Spearman’s correlation coefficient between maximal aortic valve gradient measured at 6 months and mean [^18^F]F-sodium fluoride uptake of top segment corrected by mediastinal blood pool structures uptake assessed at 6 months after TAVI procedure.

We observed a significant correlation between mean and maximal aortic valve gradient measured at baseline after the TAVI procedure and the value of Hounsfield units assessed via CT 6 months after this process (respectively: *r* = 0.32, *p* = 0.04; Figure 5A, *r =* 0.32, *p* = 0.04; Figure 5B). Additionally, significant correlations were found between maximal aortic valve gradient at 6 months and mean [^18^F]F-NaF uptake of the top segment corrected by mediastinal blood pool structures uptake assessed at 6 months after TAVI procedure (*r* = −0.4, *p* = 0.04, Figure 5C).

#### 3.4.3. Valve Type

**Figure 6:**
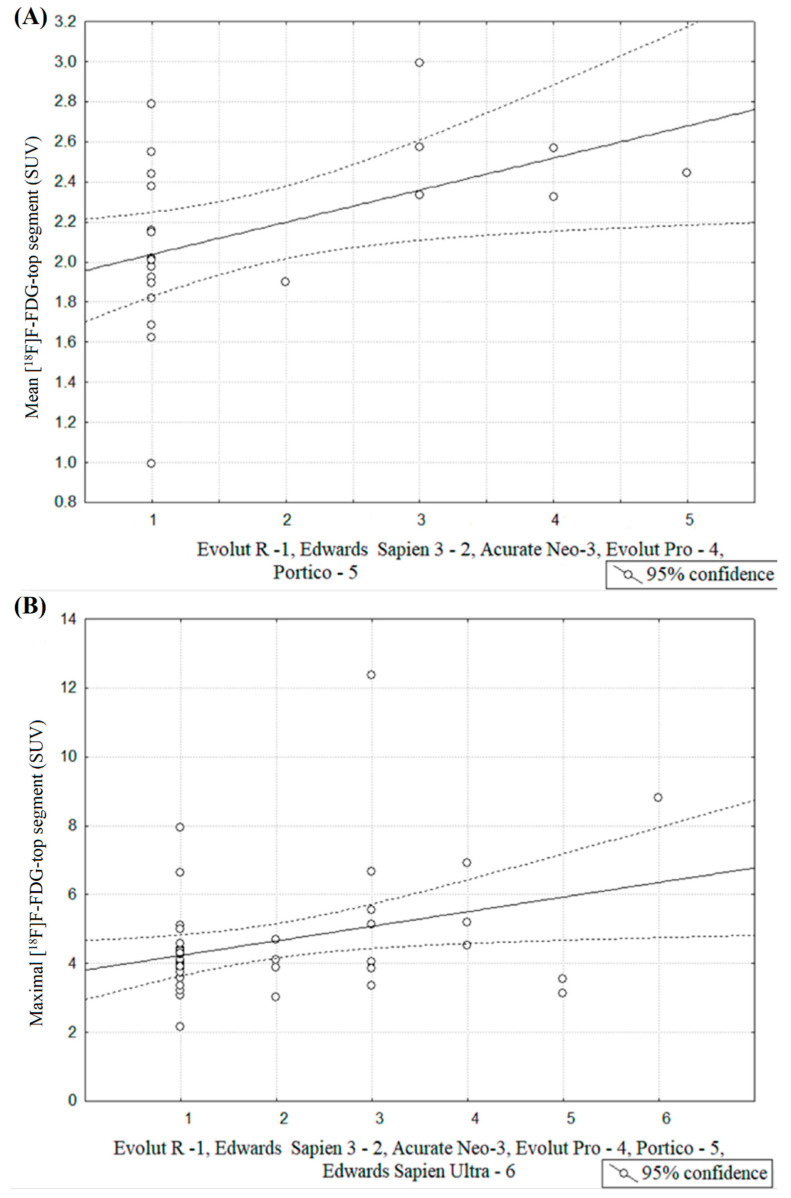
(**A**) Spearman’s correlation coefficient between valve type and mean [^18^F]F-fluorodeoxyglucose top segment measured at baseline after TAVI procedure. (**B**) Spearman’s correlation coefficient between valve type and maximal [^18^F]F-FDG top segment measured at baseline after TAVI procedure.

We noted a significant relationship between valve type and mean [^18^F]F-FDG SUV for the top segment (*r* = 0.46, *p* = 0.03), which was the lowest for the Evolut R, and respectively higher for the Edwards Sapien 3, Acurate Neo, Evolut Pro and the highest for Portico (Figure 6A). A similar relationship was found between the valve type and maximal [^18^F]F-FDG SUV for the top segment (*r* = 0.32, *p* = 0.03), while it was the lowest for Evolut R, and higher for Edwards Sapien 3, Accurate Neo, Evolut Pro, Portico and the highest for Edwards Sapien Ultra, respectively (Figure 6B). In contrast, there no significant correlations between the valve type and other PET parameters.

#### 3.4.4. TAVI Valve Insufficiency

There were no significant correlations between the grade of TAVI valve insufficiency assessed by echocardiography and maximal, mean and corrected by MBPS (TBR) uptakes for inner and outer mapping areas assessed by [^18^F]F-FDG and [^18^F]F-NaF.

## 4. Discussion

The main findings of the current study are significant positive correlations of valve types and mean, as well as maximal [^18^F]F-FDG SUV for the top segments. and significant differences between inner and outer assessment areas for maximal [^18^F]F-NaF and [^18^F]F-FDG SUV, and those corrected by MBPS (TBR), independently of segment of mapping. Significant negative correlations were noticed between EOA and transaortic gradients assessed during FU after the TAVI procedure, as well as Hounsfield unit values evaluated via CT and maximal [^18^F]F-NaF SUV of the bottom segment.

At present, the durability of TAVI valves is a crucial question because of gradually expanding indications. Echocardiography is still the most frequent method used to assess morphology and function of artificial valves, including TAVI, but usually only TEE allows to evaluate morphology of the implanted bioprosthesis. However, echocardiography enables only a semi-quantitative assessment of the calcification. CT identifies macroscopic deposits of calcium with a diameter between 200 and 500 μm [7,8], while the [^18^F]F-NaF signal is higher in areas of microcalcification (<50 μm). Recently developed calcium, adjacent or remote to macro-calcific deposits will pass through this microcalcification stage so that [^18^F]F-NaF effectively provides a marker of calcification activity [9]. The association between cardiovascular [^18^F]F-NaF PET uptake and the histological staining of hydroxy apatite has been demonstrated, using excised atherosclerotic plaques [10]. It has been confirmed that [^18^F]F-NaF predominantly binds with hydroxyapatite, whereas in the aortic valve, [^18^F]F-NaF has demonstrated a close association with areas of tissue with positive histochemical staining for alkaline phosphatase and areas with positive immunohistochemical reactivity for osteocalcin [9,11]. It has also been found that [^18^F]F-NaF binding is increased in regions of microcalcification, rather than large macroscopic deposits. Summarizing, [^18^F]F-NaF detects microcalcifications and areas of calcification activity, whereas CT detects established macroscopic deposits of calcium [12]. Calcification appears to play a crucial role in bioprosthetic valve degeneration, acting as a major pathological contributor to both progressive bioprosthetic valve narrowing and leaflet tears. It has been depicted ex vivo using [^18^F]F-NaF PET/CT in bioprosthetic aortic valves that leaflet uptake correlates with micro-calcific and macro-calcific deposits within the valve leaflets and colocalized with regions of tissue degradation, pannus and thrombus on histological examination [2]. Similar to the ex vivo findings, increased [^18^F]F-NaF uptake colocalized with areas of spotty calcification, non-calcific leaflet thickening and pannus have been observed on the CT. Patients with increased [^18^F]F-NaF uptake at baseline demonstrated clear evidence of deteriorating bio-prothesis function after two years, whereas patients without uptake, presented no change in valve function during the same observational period of time. However, the native valve degeneration process is initiated by local inflammatory processes, and other markers can be used to illustrate this stage of degeneration. [^18^F]F-FDG is a PET tracer, the uptake of which can be used to measure metabolic activity in the aortic valve as a marker of inflammation [13]. Dweck et al. recruited 121 patients with a range of calcific aortic valve disease who underwent both [^18^F]F-FDG and [^18^F]F-NaF PET/CT imaging [13]. [^18^F]F-NaF uptake activity was higher in aortic stenosis patients than controls, and increased progressively with more advanced stages of aortic stenosis. Interestingly, increased [^18^F]F-FDG activity was also detected, but uptake values were lower than [^18^F]F-NaF and had only a mild association with disease severity. Particularly, in more advanced stages of the disease, calcification, rather than inflammation, had a more predominant role in disease progression [14]. Based on the research above, we planned to evaluate the use of PET/CT to illustrate the precursory degenerative abnormalities beginning from local inflammation and microcalcifications. The first PET/CT, sixmonths after implantation, was mainly aimed to obtain the baseline characteristics of the TAVI valve and adjacent tissue uptake so that the data could be compared to the value in the two-year follow-up. Others, when analyzing the uptake of degenerate bioprostheses, applied analysis of a circular region with a 1-cm diameter. Taking the pathomechanism of the native aortic valve degeneration into account, starting from the base of the leaflets towards their margins, and the characteristics of our study group (we do not expect significant degeneration during the six-month and two-year follow-up), we decided to use the inner contour of the TAVI valve stent on three levels so as to cover the largest possible surface of the leaflets, including their base. Currently, no data are available on the pathomechanism of TAVI degeneration apart from only a few case reports. On this basis, we think this should be the target of future therapeutic interventions, although it must be acknowledged that other processes, such as fibrosis, also determine valve stiffening and progression. Moreover, [^18^F]F-NaF PET predicts where new areas of calcium would develop in CT on repeated scans performed two years later, consistent with it acting as a marker of calcification activity and, therefore, providing powerful prediction of disease progression and future aortic valve replacement or cardiovascular mortality [15]. Additional evidence has demonstrated that [^18^F]F-NaF is a more sensitive and specific biomarker than [^18^F]F-FDG in the assessment of atherosclerotic disease, and this has been also confirmed for aortic stenosis [16]. We believe that echocardiography remains the first-line method in the assessment of bioprostheses function, including hemodynamics, so important for invasive therapy qualification. However, confirmation of the possibility to assess early degenerative changes concerning implanted valves in the PET/CT will, on the one hand, broaden our knowledge on the pathomechanism, and, on the other, possibly open a new way to precisely investigate the pharmacotherapeutic efficacy of this process.

The TBR values obtained in the present study seem to be comparable to those provided in other publications. The mean values for the averaged internal and external areas in the presented publication are indeed comparable to the values published in other works, e.g., in that published by Joshi et al., in which coronary arteries were examined within the context of vulnerable atherosclerotic plaque presence (median TBR 1.66 for culprit plaques and 1.24 for non-culprit plaques), or in the work published by Cartilage et al., in which bioprosthetic aortic valves were tested and found to have mean TBR values of 1.48 in the case of spotty calcifications, 3.26 in the case of large calcifications or 1.39 in the case of non-calcific leaflet thickening [2,17]. However, in the present study, freshly implanted biological valves were assessed without degenerative changes or calcifications. Therefore, it may be presumed that the expected TBR values in the current study will be somewhat lower, nonetheless, we analyzed a larger ROI area than Cartilage et al., which can be the explanation for higher baseline values.

In the previously reported publications, SUV MBPS measurements were taken at the outlet of the right coronary artery level, which was not possible in our study due to the presence of numerous calcifications as remnants of previous valve and structural components of the newly implanted valve. Therefore, in order to establish the measurement methodology, we made an estimated measurement in the descending aorta, at the level of the implanted valve. Surgical procedures enable the removal of calcification during the aortic valve replacement procedure, while the transcatheter procedure does not. In the case of TAVI implantation, the calcified native leaflets remain outside the valve stent, but this can potentially influence the uptake results. Therefore, in order to investigate their influence on the SUV uptake measurements at the leaflet level, we performed uptake analysis for the inner area including the TAVI leaflets of the implanted valve itself and for the outer area also including the wider circle with the above-mentioned structures. Measurements were also carried out depending on the SUV level for bottom, medium and top uptakes. As expected, we showed significantly higher SUV and TBR values for outer area when compared to the inner one, but only for maximal SUV and TBR, but not for mean, regardless of the marking level. TBR values were comparable to those reported in the literature, however, they were higher than expected. This could be the result of signal interference from outer regions as well as calcifications of native rings and leaflets, or larger analyzed area [2,17].

As for the inter-relationships between Hounsfield values at the six-month FU and echocardiographic parameters (EOA) [2], this may certainly be explained by the fact that the larger calcifications of native valve and annulus can influence the measurement of attenuation (HU), especially in the case of small TAVI valve size (with smaller area), and on the other hand, sometimes large annulus and valve calcification. Furthermore, the border size of the annulus necessitate the choice of a smaller valve size during the TAVI procedure.

For the same reason, we showed a positive correlation between the transaortic valve gradient measured at baseline (after TAVI procedure) and the leaflet attenuations (HU) [^18^F]F-FDG uptake. Nevertheless, we have also shown that the mean (inner and outer) TBR of [^18^F]F-NaF measured over the top segment correlated negatively with transaortic valve gradient measured at baseline (after TAVI procedure). Perhaps this is a coincidence, because for other levels, the pre-correction values of MBPS, maximal values and [^18^F]F-FDG uptakes have not been shown to be significant.

However, when considering the relationship between PET uptakes and valve types, according to our knowledge, such relationships have not been described so far in the literature. It is obvious that this may undoubtedly be a consequence of the different construction of TAVI valves, especially their scaffolding. In our publication, we showed that the smallest SUV uptakes for mean and maximal [^18^F]F-FDG top segments were related to the Evolut R valve, while the largest, to Sapien 3 Ultra and Portico. The results should be interpreted with caution, nevertheless, it can be concluded that the potential norms and where the SUV is measured may differ depending on valve type.

### Limitations

The observation period in the present study is short, the last PET is to be performed two years after the TAVI procedure, thus, perhaps this period will not be long enough for the appearance of degenerative changes detectable by PET/CT. A significant limitation is the potential influence of stents on CT and PET measurements.

## 5. Conclusions

PET/CT imagining with the use of [^18^F]F-FDG and [^18^F]F-NaF is intended to be feasible, and it practically allows the standardized uptake value (SUV) to differentiate the area containing the TAVI leaflets from the SUV directly adjacent to the ring calcifications and the calcified native leaflets. This could become the seed for future detection and evaluation capabilities of progression regarding even early TAVI valve degenerative lesions, expressed as local leaflet inflammation and micro-calcifications.

### Impact on Daily Practice

Defining predictors of early degeneration may enable identifying a group of patients more exposed to earlier bioprosthesis dysfunction, which may be crucial, especially in terms of more frequent TAVI implantation in the younger population. The results of this study may be useful for the development of new therapeutic strategies targeted at improving the durability of implanted aortic valves (e.g., pharmacological treatment), and in the future, may help design and construe the next generation of valves.

## Figures and Tables

**Table 1:** Initial clinical characteristics.

Selected Indices	(*n* = 73)
Age, years	82.49 ± 7.11, 85 (80 ÷ 87)
Gender, males	31 (42.4)
Body mass, kg	72.05 ± 14.77, 71 (60 ÷ 80)
Body height, cm	163.68 ± 9.58, 165 (158 ÷ 170)
Body-mass index, kg/m^2^	26.80 ± 4.51, 26.45 (23.05 ÷ 29.86)
Body Surface area, m^2^	1.78 ± 0.21, 1.77 (1.63 ÷ 1.95)
Glomerular filtration rate (GFR), mL/min./1.73 m^2^	62.69 ± 18.85, 64 (48 ÷ 78)
NYHA classification before TAVI:—I	3 (4.1)
—II	15 (20.5)
—III	50 (68.5)
—IV	5 (6.9)
Kidney failure (GFR < 60 mL/min./1.73 m^2^)	31(42.3)
Arterial hypertension	65(90.1)
Diabetes mellitus	28(39.4)
Atrial fibrillation	38 (52.1)
Prior myocardial infarction	24(35.2)
Prior percutaneous coronary interventions	29 (40.8)
Prior coronary artery by-pass surgery	12 (16.9)
Prior balloon aortic valvuloplasty	27 (38.0)
Carotid artery stenosis	4 (5.6)
Chronic obstructive pulmonary disease	10 (14.1)
Prior cerebral stroke/transient ischaemic attack	10 (14.1)
Prior pacemaker implantation	12 (16.9)

Data are presented as arithmetic mean ± standard deviation, median (lower ÷ upper interquartile range) and numbers (percentages). NYHA, New York Heart Association.

**Table 2:** Initial and follow-up echocardiographic parameters.

Selected Indices	Before TAVI (Initial)	After TAVI (Baseline)	Follow-Up	
			1 Month	6 Months
Mean gradient before TAVI, mmHg	52.93 ± 19.13	9.42 ± 3.81	9.95 ± 3.83	10.06 ± 3.89
	50 (40 ÷ 66)	9 (7.29 ÷ 11.07) *	9.82 (7.44 ÷ 11.77) *	9.46 (7.54 ÷ 12.11) *
Peak gradient after TAVI, mmHg	17.55 ± 7.10		17.97 ± 6.23	18.04 ± 6.41
	17.5 (13.9 ÷ 20.54)		17.88 (13.47 ÷ 20.52)	17.29 (13.75 ÷ 21.1)
Effective orifice area, cm^2^	0.63 ± 0.17		1.52 ± 0.32	1.74 ± 1.51
	0.6 (0.5 ÷ 0.7)		1.5 (1.3 ÷ 1.7)	1.5 (1.32 ÷ 1.77)
Left ventricle ejection fraction, %	52.10 ± 15.34	54.74 ± 12.53	57.43 ± 12.54	56.79 ± 11.47
	60 (40 ÷ 65)	60 (45 ÷ 65)*	60 (50 ÷ 66)*	60 (48.75 ÷ 65)*
Aortic regurgitation				
—none	5 (7.1)	42 (57.4)	49 (92.4)	51 (87.9)
—trace	15 (20.5)	19 (26.2)	4 (7.6)	6 (10.4)
—mild	29 (39.5)	12 (16.4)	0 (0)	1 (1.7)
—moderate	16 (22.5)	0 (0)	0 (0)	0 (0)
—severe	8 (11.2)	0 (0)	0 (0)	0 (0)
Paravalvular leak grade			1.69 ± 1.32	1.62 ± 1.33
			1 (1 ÷ 2)	1 (1 ÷ 2)
—none			7 (13.2)	10 (17.24)
—trace			11 (20.75)	11 (18.96)
—mild			24 (45.28)	25 (43.1)
—moderate			11 (20.75)	11 (18.96)
—severe			0 (0)	1 (1.72)

Data are presented as arithmetic mean ± standard deviation, median (lower ÷ upper interquartile range) and numbers (percentages). *—*p* < 0.05 when selected indices after TAVI were compared to those assessed before TAVI CT, computed tomography; TEE, transesophageal echocardiography; TAVI, transcatheter aortic valve implantation.

**Table 3:** Positron emission tomography-computed tomography.

Assessment Area	Indicator Type	Marker Type					
		[^18^F]F-Sodium Fluoride			[^18^F]F-Fluorodeoxyglucose		
		Mapping Level					
		Top	Middle	Bottom	Top	Middle	Bottom
SUV inner	Mean	1.89 ± 0.48	1.89 ± 0.62	1.87 ± 0.53	2.16 ± 0.5	2.18 ± 0.43	2.17 ± 0.42
		1.95	1.84	2.02	2.27	2.1	2.13
		(1.57 ÷ 2.2)	(1.44 ÷ 2.38)	(1.47 ÷ 2.35)	(1.84 ÷ 2.43)	(1.92 ÷ 2.54)	(2 ÷ 2.5)
	Mean—TBR	1.55 ± 0.52	1.57 ± 0.64	1.54 ± 0.56	1.59 ± 1.1	1.54 ± 0.58	1.57 ± 0.75
		1.47	1.55	1.47	1.34	1.43	1.5
		(1.11 ÷ 1.93)	(1.0 ÷ 1.86)	(1.25 ÷ 1.79)	(1.13 ÷ 1.6)	(1.13 ÷ 1.71)	(1.1 ÷ 1.85)
	Max.	4.15 ± 1.35 *	4.39 ± 1.42 *	4.24 ± 1.33 *	4.41 ± 1.77 *	4.4 ± 1.32 *	4.35 ± 1.24 *
		3.95	4.09	4.22	3.9	4.02	4.17
		(3.36 ÷ 5.18)	(3.55 ÷ 5.07)	(3.47 ÷ 5.03)	(3.46 ÷ 4.96)	(3.6 ÷ 4.98)	(3.61 ÷ 4.82)
	Max. TBR	3.02 ± 1.21 *	3.23 ± 1.4 *	3.14 ± 1.38 *	2.98 ± 3.28	2.89 ± 2.38	2.85 ± 1.99
		2.66	2.98	3.06	2.44	2.56	2.39
		(2.05 ÷ 3.84)	(2.26 ÷ 3.84)	(2.18 ÷ 3.76)	(2.01 ÷ 2.99)	(2.06 ÷ 2.99)	(1.93 ÷ 3.26)
SUV outer	Mean	1.95 ± 0.55	1.99 ± 0.63	1.88 ± 0.52	2.15 ± 0.42	2.25 ± 0.4	2.2 ± 0.42
		1.92	2.05	1.86	2.04	2.22	2.29
		(1.58 ÷ 2.29)	(1.58 ÷ 2.62)	(1.52 ÷ 2.29)	(1.95 ÷ 2.43)	(1.98 ÷ 2.52)	(1.97 ÷ 2.43)
	Mean—TBR	1.61 ± 0.62	1.66 ± 0.74	1.56 ± 0.63	1.54 ± 0.65	1.63 ± 0.78	1.59 ± 0.73
		1.48	1.52	1.41	1.49	1.55	1.52
		(1.13 ÷ 1.86)	(1.15 ÷ 1.96)	(1.14 ÷ 1.85)	(1.1 ÷ 1.77)	(1.14 ÷ 1.84)	(1.09 ÷ 1.81)
	Max.	5.37 ± 1.71	5.33 ± 1.71	5.32 ± 1.96	4.82 ± 1.68	5.09 ± 2.06	5.36 ± 2.32
		4.99	5.19	5.09	4.41	4.59	4.84
		(4.16 ÷ 6.34)	(4.14 ÷ 6.02)	(3.92 ÷ 6.22)	(3.88 ÷ 5.31)	(3.99 ÷ 5.48)	(4.21 ÷ 5.74)
	Max.—TBR	3.92 ± 1.53	3.93 ± 1.76	3.91 ± 1.79	3.26 ± 3.31	3.54 ± 4.31	2.55 ± 1.02
		3.77	3.66	3.52	2.78	2.78	2.35
		(2.63 ÷ 4.91)	(2.62 ÷ 4.72)	(2.83 ÷ 4.72)	(2.21 ÷ 3.37)	(2.29 ÷ 3.38)	(2.01 ÷ 2.82)

Data are presented as arithmetic mean ± standard deviation and median (lower ÷ upper interquartile range). * *p* < 0.05 when the inner and outer areas of assessment were compared for particular mapping levels and indicator types; SUV, standardized uptake value; TBR, tissue-to-background ratio.

## Data Availability

The data presented in this study are available on request from the corresponding author.
